# Genomic Analysis of Prophages from *Klebsiella pneumoniae* Clinical Isolates

**DOI:** 10.3390/microorganisms9112252

**Published:** 2021-10-28

**Authors:** Andreia T. Marques, Luís Tanoeiro, Aida Duarte, Luisa Gonçalves, Jorge M. B. Vítor, Filipa F. Vale

**Affiliations:** 1 Pathogen Genome Bioinformatics and Computational Biology, Research Institute for Medicines (iMed-ULisboa), Faculty of Pharmacy, Universidade de Lisboa, 1649-003 Lisboa, Portugal; luistanoeiro@gmail.com (L.T.); jvitor@ff.ulisboa.pt (J.M.B.V.); 2 Faculty of Pharmacy, Universidade de Lisboa, Av. Gama Pinto, 1649-003 Lisboa, Portugal; duarte.aida5@gmail.com; 3 Centro de Investigação Interdisciplinar Egas Moniz, Instituto Universitário Egas Moniz, 2829-511 Monte da Caparica, Portugal; 4 Clinical Pathology Unit, Hospital SAMS, Cidade de Gabela, 1849-017 Lisboa, Portugal; mariallgodinho@gmail.com

**Keywords:** *K. pneumoniae* genomes, prophages, bacteriophage, bioinformatics, genomic analysis, comparative genomics, phylogeny, sequence annotation and comparison, phage endolysins

## Abstract

*Klebsiella pneumoniae* is an increasing threat to public health and represents one of the most concerning pathogens involved in life-threatening infections. The resistant and virulence determinants are coded by mobile genetic elements which can easily spread between bacteria populations and co-evolve with its genomic host. In this study, we present the full genomic sequences, insertion sites and phylogenetic analysis of 150 prophages found in 40 *K. pneumoniae* clinical isolates obtained from an outbreak in a Portuguese hospital. All strains harbored at least one prophage and we identified 104 intact prophages (69.3%). The prophage size ranges from 29.7 to 50.6 kbp, coding between 32 and 78 putative genes. The prophage GC content is 51.2%, lower than the average GC content of 57.1% in *K. pneumoniae*. Complete prophages were classified into three families in the order *Caudolovirales*: *Myoviridae* (59.6%), *Siphoviridae* (38.5%) and *Podoviridae* (1.9%). In addition, an alignment and phylogenetic analysis revealed nine distinct clusters. Evidence of recombination was detected within the genome of some prophages but, in most cases, proteins involved in viral structure, transcription, replication and regulation (lysogenic/lysis) were maintained. These results support the knowledge that prophages are diverse and widely disseminated in *K. pneumoniae* genomes, contributing to the evolution of this species and conferring additional phenotypes. Moreover, we identified *K. pneumoniae* prophages in a set of endolysin genes, which were found to code for proteins with lysozyme activity, cleaving the β-1,4 linkages between N-acetylmuramic acid and N-acetyl-D-glucosamine residues in the peptidoglycan network and thus representing genes with the potential for lysin phage therapy.

## 1. Introduction

*Klebsiella pneumoniae* is an opportunistic and commensal gram-negative human pathogen prevalent in the hospital environment. This bacterium is mainly found in gastrointestinal and respiratory tracts and on the skin of healthy individuals, but in recent years it has become one of the world’s leading causes of community and hospital-acquired infections, such as urinary tract infections (UTIs), pneumonia, septicaemia, and wound/soft tissue infections, with an increasing mortality rate, particularly in immunocompromised individuals, neonates, and the elderly [1,2,3,4,5].

Due to its widespread distribution and genetic plasticity, *K. pneumoniae* is one of the most important multidrug-resistant (MDR) pathogens and has been classified as an ESKAPE organism (*Enterococcus faecium*, *Staphylococcus aureus*, *K. pneumoniae*, *Acinetobacter baumannii*, *Pseudomonas aeruginosa* and *Enterobacter species*) [6], in which antibiotic-resistant strains are becoming more difficult to treat. *K. pneumoniae* strains are recurrently resistant to antibiotics available in therapy to treat serious human diseases, such as fluoroquinolones, aminoglycosides, and beta-lactams. Among beta-lactams, penicillins, cephalosporins and carbapenems, there is increasing evidence of infections caused by strains that have become resistant to imipenem, ertapenem and meropenem antibiotics [5,7,8,9,10,11]. Given the reduction in the effectiveness of antimicrobial therapeutics to treat *K. pneumoniae*-associated infections, alternative strategies must be developed in response.

Bacteriophages (phages) are viruses that infect bacteria. Viruses were initially suggested as the first antimicrobial agents by William Twort and Felix d’Herelle [12,13] and were a therapy for bacterial infections [14]. However, after the discovery of antimicrobial compounds, phage therapy was discarded (except in Eastern Europe and the former Soviet Union [15]), and from there on most studies addressed prophage research as a tool to improve our understanding of molecular biology, horizontal gene transfer and bacterial evolution.

More recently, given the increase in the number of MDR infections caused by gram-negative bacteria such as *K. pneumoniae*, the use of phages or specific phage gene products has increased again as a potential alternative to current antimicrobial therapies [16]. MDR isolates of *K. pneumoniae* were found with a variable number of prophages in their chromosomes [17], and some prophages carried antibiotic resistance genes (ARGs) [18,19], prompting the interest in bacteriophage research.

Phages of *K. pneumoniae* have been isolated from a variety of sources worldwide, including wastewater, sewage, seawater, and human intestinal samples. Such phages belong to four of the five families of the order *Caudovirales*, described as non-enveloped, tailed phages, with icosahedral heads containing double-stranded DNA: *Myoviridae* are characterized by long, straight, contractile tails; *Siphoviridae* by long, flexible, non-contractile tails; *Podoviridae* by short, non-contractile tails; and *Ackermannviridae* by contractile tails with up to four spikes present on each of six tail spike entities [20].

Phages are a group of viruses that infect bacteria and make use of bacterial replication machinery to become replicated, generating progeny and releasing it in the environment mostly by promoting the cell–host lysis in the lytic cycle [21,22]. In the lysogenic cycle, phages can integrate in the host genome and remain dormant for an unspecified amount of time as prophages, which will be replicated as part of the bacterial genome without killing the host. Under certain conditions (e.g., in the presence of environmental stressors), prophages can be excised and induced, assuming a lytic cycle, and begin actively replicating and producing viable phage particles [23]. In addition, during the lysogenic cycle, genetic changes may happen in prophage sequences, which lead to cumulative degradation of the bacteriophage genome or transference of genes into the host that can confer toxin production and antibiotic resistance traits to the bacterium genome, thus making the infection more virulent and difficult to treat [24]. Likewise, prophages can contribute to important biological properties of their bacterial hosts, such as fitness, virulence, and evolution [21]. Even defective prophages may provide multiple benefits to the host for surviving adverse environmental conditions [25]. 

Endolysins (lysins) are peptidoglycan-hydrolyzing enzymes encoded by phage genes. In the cell disruption stage of the phage lytic cycle, lysins are involved in the breakage of peptidoglycan to release phage progeny [26]. In the case of gram-negative bacteria, peptidoglycan has a highly conservative structure with significant similarities shared among different species; therefore, endolysins are usually active against a wide host range [27,28]. Moreover, endolysins feature reasonable selectivity in targeting pathogenic species, preserving commensal microflora, and the administration of these phage-derived enzymes can be easily applied by different strategies, including parenteral, topical or oral formulations [27]. For these reasons, the use of endolysins is more attractive as an alternative antimicrobial agent for clinical treatment than the phage itself; therefore, lysins have been proposed as alternative antimicrobial agents to treat infections in the post-antibiotic era [29,30,31]. Recent research has produced some promising results regarding the use of endolysins against *K. pneumoniae*. A recombinant endolysin from the *K. pneumoniae* phage KP27 was produced, and its peptidoglycan-degrading activity was demonstrated against gram-negative bacteria by the co-incubation of bacteria and endolysin [32]. In a study by Walmagh et al. (2013), five endolysins were characterized, including two endolysins from *K. pneumoniae* phages K11 and KP32, and their muralytic activity on the peptidoglycan of several gram-negative bacterial species was demonstrated [33]. In another study, two endolysins (ElyA1 and ElyA2) combined with colistin were tested against *A. baumannii*, *P. aeruginosa* and *K. pneumoniae*, and one of them displayed activity against 13 out of 17 strains of *K. pneumoniae* [34].

In this work, we aimed to evaluate the prophage presence in clinical isolates of *K. pneumoniae* from an outbreak in a Portuguese tertiary-care hospital. Also, we aimed to understand how prophages can contribute to the rapid evolution of this bacterial pathogen. Moreover, we have identified and characterized putative endolysin genes encoded by these prophage genomes that can potentially be used for phage lysin therapy.

## 2. Materials and Methods

### 2.1. K. pneumoniae Isolates Genomes

A total of 40 multiclonal *K. pneumoniae* isolates from 23 patients hospitalized in intensive care unit at SAMS Hospital, a Portuguese tertiary-care hospital, were recently sequenced by whole genome sequencing (WGS) [35] and the genomes were screened for prophage presence.

### 2.2. Prophage Identification

PHASTER (PHAge Search Tool Enhanced Release) [36] and Prophage Hunter Tool [37] were used, allowing for the identification and annotation of putative prophages within contigs of each *K. pneumoniae* genome (last accessed January 2021). Concurrently, bacterial genomes were also annotated using the open-access tool RAST: Rapid Annotation using Subsystem Technology [38,39,40], and the identified prophage genes were extensively analysed in terms of sequence and structure to evaluate its homology with bacteriophage-derived regions. The annotation of prophage coding sequences found by the three different methods was compared (data not shown).

All prophage sequences were manually sorted and curated, and the insertion sites were determined as shown below. A complete prophage sequence is not often present in one contig, and to overcome this limitation, whenever possible, prophages were scaffolded using BLAST with a query of the prophages from *K. pneumoniae* subsp. *pneumoniae* HS11286 (GenBank Accession: CP003200.1, genome region from 1288358 to 1338717) and *K. pneumoniae* strain FDAARGOS_775 (GenBank Accession: NZ_CP040993.1, genome region from 3328442 to 3378114) to check for homologies in the contigs in a similar way, as described elsewhere [41]. Both genomes were available on NCBI database (https://www.ncbi.nlm.nih.gov/, last accessed 15 January 2021) as reference genomes for *K. pneumoniae* and, since both have integrated prophages, we also referred to them to determine the correct insertion sites. The insertion sites of the prophages were identified whenever the prophage 5′ and 3′ ends were contiguously flanked by bacterial genes in a contig. The last bacterial gene before the prophage sequence and the first bacterial gene after the prophage sequence were identified.

Each annotated region was analysed in terms of nucleotide sequence with BLASTn [42] and phage-limited BLASTn (limited to bacteriophage-related tax ids: 38018, 10699, 10662, 10744, 10841, 2100421, 28883, 12333, 79205 and 102294) in the NCBI database using default parameters. Protein-coding sequences were also analysed using regular BLASTp and phage-limited BLASTp (limited to bacteriophage-related tax ids: 38018, 10699, 10662, 10744, 10841, 2100421, 28883, 12333, 79205 and 102294). Structural homology analyses were performed using Phyre2 [43].

### 2.3. Prophage Classification

The identified intact prophages were classified in silico into their respective phage families based on the prophage structural head-neck-tail proteins using VIRFAM [44]. The prophage lifestyles were classified using PHACTS [45] and were additionally assessed by manually inspecting the genomes for genes related to lifestyle (e.g., integrases).

### 2.4. Prophage Pan-Genome 

The core- and pan-genome of *K. pneumoniae* intact prophages were determined using Roary (version 3.13) [46], using as settings for core genome the genes present in at least 50% of the prophage intact genomes, a minimum BLASTp percentage identity of 40, 50, 60, 70, 80 or 90%, and −s option. These settings were used to determine the most suitable parameters for determining the prophage pan-genome, as previously described [47].

### 2.5. Prophage Phylogenetic Analysis 

Intact prophage sequences were queried against all *K. pneumoniae* phages sequences available on the PATRIC website (https://www.patricbrc.org, last accessed January 2021) [48], which had 256 sequences in January 2021, and against public databases using phage-limited BLASTn [42] to identify similar phages. Hits with a query cover of at least 50% were considered similar phages and those with query covers below 50% were considered close phages.

The prophage genomes were aligned using MAFFT version 7 [49] default options. Maximum likelihood phylogenetic trees from the alignments were produced using FastTree 2.1.11 [50]. The produced trees were visualized and annotated using Interactive Tree Of Life (iTOL) v6 [51].

### 2.6. Prophage-Associated Virulence Factors and Antibiotic Resistance Genes

All prophage genomic sequences were screened for antibiotic resistance genes using the ResFinder 4.1 database (https://cge.cbs.dtu.dk/services/ResFinder-4.1/, last accessed July 2021) and virulence genes using VirulenceFinder 2.0 (https://cge.cbs.dtu.dk/services/VirulenceFinder/, last accessed July 2021). Similarly, the Resistance Gene Identifier (RGI) option of The Comprehensive Antibiotic Resistance Database (https://card.mcmaster.ca/home, last accessed July 2021) was used with default values to identify resistance genes, their products, and associated phenotypes harbored by integrated prophages within *K. pneumoniae* strains.

### 2.7. Endolysins Identification, Gene Ontology Analysis and Functional Annotation

Since defective prophages can also harbor lysins, we considered all prophages identified (intact and defective) for endolysins identification. Together with our prophage sequences, we also analysed a set of 17 annotated phages identified during prophage phylogenetic analysis, which share homology with our prophages. A total of 167 prophage sequences (150 sequences originally identified + 17 phage annotated sequences) were submitted to bioinformatic analysis for the identification of putative phage endolysins in terms of sequence homology using BLAST [42] and structural homology using the open-access tools Phyre2 [43] and SWISS-MODEL [52]. Gene Ontology (GO) identifiers and related GO terms were assigned to the identified endolysins using the QuickGo web server (http://www.ebi.ac.uk/QuickGO/, last accessed July 2021).

### 2.8. Endolysin Phylogenetic Analysis

Endolysin genomic and proteomic sequences were aligned using MAFFT version 7 [49] with default parameters. The genome phylogenetic tree was constructed using the Jukes–Cantor substitution model and the proteome phylogenetic tree was constructed using the Le Gascuel substitution model in PHYML 3.3.20180621 (Geneious Prime version 2021.1.1). The identity matrix generated during the construction of the phylogenetic trees was used to infer nucleotides and proteins endolysins identity. Trees were visualized and annotated using Interactive Tree Of Life (iTOL) v6 [51].

## 3. Results

### 3.1. Identification and Prevalence of Prophages in K. pneumoniae Strains

In the present study, the genome sequences of 40 *K. pneumoniae* clinical isolates from 23 patients were analysed with a web server tool for identification and annotation of prophage sequences within bacterial genomes, PHASTER, with default arguments [36]. 

**Figure 1:**
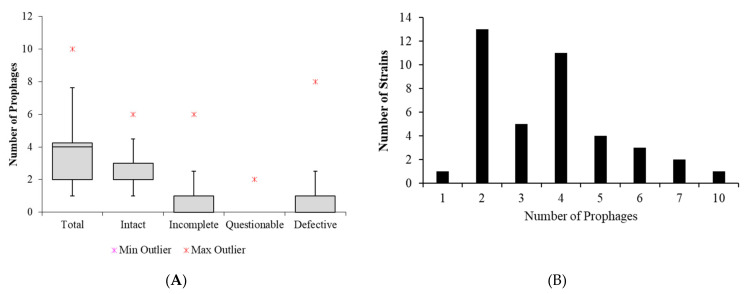
(**A**) Box plot showing the total, intact, incomplete, questionable, and defective (incomplete + questionable) prophages of *Klebsiella pneumoniae*. (**B**) Bars graph showing the distribution of total prophages (intact, incomplete, and questionable) in *K. pneumoniae* strains.

A total of 150 prophage-like elements were detected (Supplementary Table S1), of which 104 were classified as intact, 39 as incomplete and 7 as questionable (Figure 1a, Supplementary Table S2). One strain was found to harbor only one prophage, while the remaining 39 strains (97.5%) have at least two prophages, which indicates that prophages are abundant in the *K. pneumoniae* genome. The total number of prophages per strain ranged from 1 to 10, with an average of 3.7 prophages per strain, and most strains harbored either two (*n* = 13) or four (*n* = 11) prophages (Figure 1b).

A significantly higher prevalence of incomplete and questionable prophages was expected since intact prophages are usually under strong selection or genetic degradation by bacteria for rapid deletion from bacterial genomes [53,54]. Instead, we found that 97.5% of the *K. pneumoniae* strains contain intact prophages. Strains isolated from patients 1, 6, 21, 23, 24, 25 and 26 contained only intact prophages, whereas strains isolated from patients 2, 3, 5, 9, 11, 14, 16, 17, 18 and 19 contained intact and incomplete prophages, and strains isolated from patients 4, 7, 8, 10, 13 and 15 contained intact, incomplete, and questionable prophages (Supplementary Table S2). Furthermore, patients were colonized with one *K. pneumoniae* strain, except patients 1, 3, 15, 17, 19, 23 and 26, which were colonized with 6, 2, 4, 5, 3, 2 and 2 strains, respectively. It is important to note that the genomes were divided into contigs, which implies that PHASTER may have underestimated the correct number of intact prophages (some were split into different contigs and identified as incomplete or questionable prophages).

### 3.2. Genome Characteristics of K. pneumoniae Prophages

The shortest (remnant) prophage sequence is 8.9 kbp, and the biggest is 60.8 kbp, with the coding sequences (CDS) number ranging from 12 to 75. The average GC% content in all 150 prophages is 52.2% (min 45.1%, max 60.2%), while the average bacterial GC% content is 57.1% (min 54.9%, max 57.4%) (Supplementary Table S1), which suggests horizontal gene transfer of the prophage region.

Genomic analysis of the upstream and downstream regions of the prophage insertion sites revealed that prophages are integrated between different coding regions: proteins involved in metabolic pathways (18.1%), tRNA genes (16.1%), transporters (11.1%), recombinant proteins (5.4%), protein synthesis (5.4%), transferases (4.7%), transcriptional regulators (4.0%), membrane proteins (2.0%), ribosome biogenesis (1.3%) and sequences showing homology with other bacterial genes (8.7%). In the case of some incomplete prophages, it was not possible to determine the insertion site (23.2%) (Supplementary Table S1). Moreover, similar prophages from different *K. pneumoniae* strains had a conserved insertion site between the same two contiguous genes of *K. pneumoniae* reference genomes (GenBank Accession: CP003200.1 and NZ_CP040993.1).

For the purpose of our analysis, incomplete and questionable prophage sequences divided into different contigs were scaffolded as described in materials and methods. After this analysis, prophages still considered questionable, and incomplete were grouped as defective prophages. Prophages smaller than 28 kbp were considered not intact because they lacked a prophage genomic structure and were difficult to distinguish from other integrative elements. Only prophages with identified integrase and/or at least one gene involved in biological processes (e.g., terminase, endolysin, capsid, tail fibers) were considered intact.

According to this criterion, prophages were found to be intact in 104 of the 150 prophage sequences (63.3%) (Table 1). Intact prophages have an average of 50 predicted genes (min 32, max 78), 37.4 kbp (min 29.7 kbp, max 50.6 kbp), and 51.2% GC (min 48.3%, max 55.0%). 

### 3.3. Classification of K. pneumoniae Prophages

**Figure 2:**
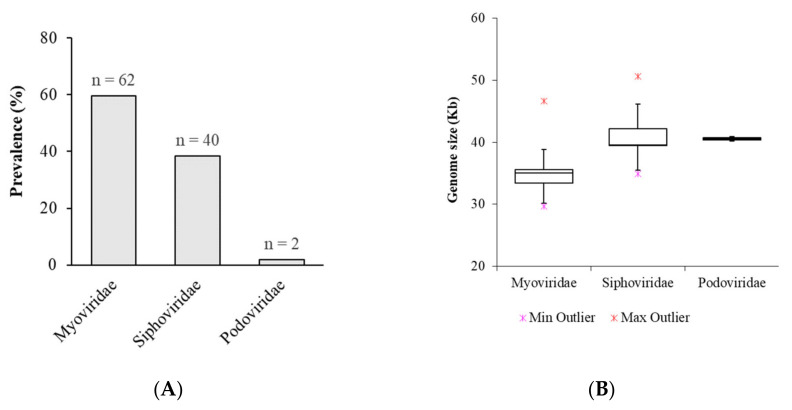
Distribution of 104 intact prophages integrating *K. pneumoniae* genomes by in silico determined family. (**A**) Prevalence of prophages in *K. pneumoniae* genomes by family. (**B**) Box plot of average genome size of prophages according to family.

Intact prophages identified were in silico assigned to a family taxon using the VIRFAM website [44]. Classification was based on genes considered to be the most indicative of its family: major capsid protein, large terminase subunit, tail tape measure protein and tail sheath protein. All prophages could be assigned to a family. The majority, 62 (59.6%) was assigned to the *Myoviridae* family, 40 (38.5%) to the *Siphoviridae* family, and 2 (1.9%) to the *Podoviridae* family (Figure 2). This is in accordance with the estimated distribution described in the literature [55,56]. Based on information on the Expansy website (http://viralzone.expasy.org/, last accessed July 2021), *Myoviridae* are typically the largest phages with a high variability in their genome sizes, ranging from 33 to 244 kbp and coding for 40 to 415 proteins. In this study, all prophages belonging to the *Myoviridae* family have genomes with an average of 34.8 kbp (min 29.7 kbp, max 46.7 kbp) and coding for 45 proteins (min 40, max 55). The *Siphoviridae* family found in *K. pneumoniae* has a genome size of around 41.3 kbp (min 35.2, max 50.6) and coding for about 56 proteins (min 32, max 78), while the described genome size of the *Siphoviridae* family is about 50 kbp and encodes for about 70 genes. Here, *Podoviridae* have the more consistent genome size of about 40.5 kbp (min 40.2 kbp, max 40.9 kbp) and coding for 55 proteins (min 53, max 57), which agrees with their usually described size around 40–42 kbp, containing about 55 genes. However, we identified only two prophages belonging to this family, which may justify lesser variability. Interestingly, all the strains colonizing each patient harbor prophages belonging to the same family, except patients 17, 24 and 25. According to Table 1, patient 17 had five isolates, of which three (Kp4874, Kp4875, Kp4876) harbored prophages belonging only to *Siphoviridae* family; the Kp4872 harbored *Siphoviridae* (*n* = 2) and *Podoviridae* (*n* = 1) prophages, and the Kp4873 harbored *Siphoviridae* (*n* = 2) and *Myoviridae* (*n* = 1) prophages. On the other hand, the isolate Kp4886 from patient 24 harbored three prophages belonging to *Siphoviridae* (*n* = 2) and *Myoviridae* (*n* = 1), and the isolate Kp4887 from patient 25 harbored *Myoviridae* (*n* = 1), *Siphoviridae* (*n* = 1) and *Podoviridae* (*n* = 1) prophages.

All prophages were predicted to have a temperate lifestyle by PHACTS, except for Kp4866-6 prophage, which was not confidently predicted to have a lytic lifestyle. However, all prophage genomes, including Kp4866-6, contained an integrase gene and a BLASTn showed that Kp4866-6 have similarity to *Klebsiella michiganensis* (up to 99.94% identity and 75% query coverage) and *K. pneumoniae* (up to 97.28% identity and 57% query coverage) genomes, could indicate that have also a temperate lifestyle.

**Figure 3:**
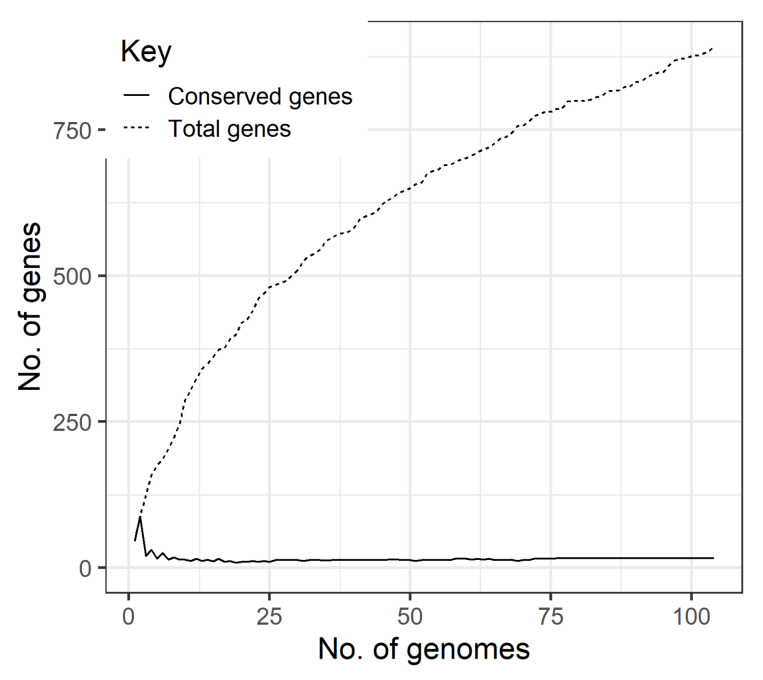
Pan-genome of *K. pneumoniae* prophages. The size of the core genome (continuous line) and pan-genome (dashed line) as more genomes are added.

Concerning the pan-genome (i.e., the entire set of genes in genomes) and the core genome (i.e., the set of genes that are present in all genomes), we found that *K. pneumoniae* prophages have an open pan-genome (Figure 3) made of 892 genes (considering a 40% identity threshold for BLASTp) or 1285 genes (if the threshold is raised to 90%). Considering the typical high genomic diversity of prophages [47], we considered the threshold of 40% for BLASTp to list the prophage pan-genome (Supplementary Table S3). Additionally, no core genes (present in at least half of the prophages) are found for thresholds of protein identity higher than 50%. However, considering the presence in at least 50% of the genomes as a core gene we could find 3 core genes if considering the protein identity threshold of 50% and 16 core genes for a threshold of 40% identity. In Supplementary Table S3, it is possible to observe that 389 genes are singletons, present in one genome only.

### 3.4. Genomic and Proteomic Phylogenetic Relationships between K. pneumoniae Prophages

The 104 intact prophage sequences were compared to a list of 256 *K. pneumoniae* phage sequences available on the PATRIC website, and we used the BLASTn [42] tool for prophage identification. Hits with a query cover of at least 50% were considered similar prophages and query covers ranging from 20% to 50% were considered close phages. Using this criterion, 13 *Klebsiella* phages were identified that were highly similar (≥50% genome homology) to our prophage sequences, as well as 4 *Klebsiella* phages and 1 *Pseudomonas* phage (VW-6B) with ≥20% genome homology (Table 1).

**Figure 4:**
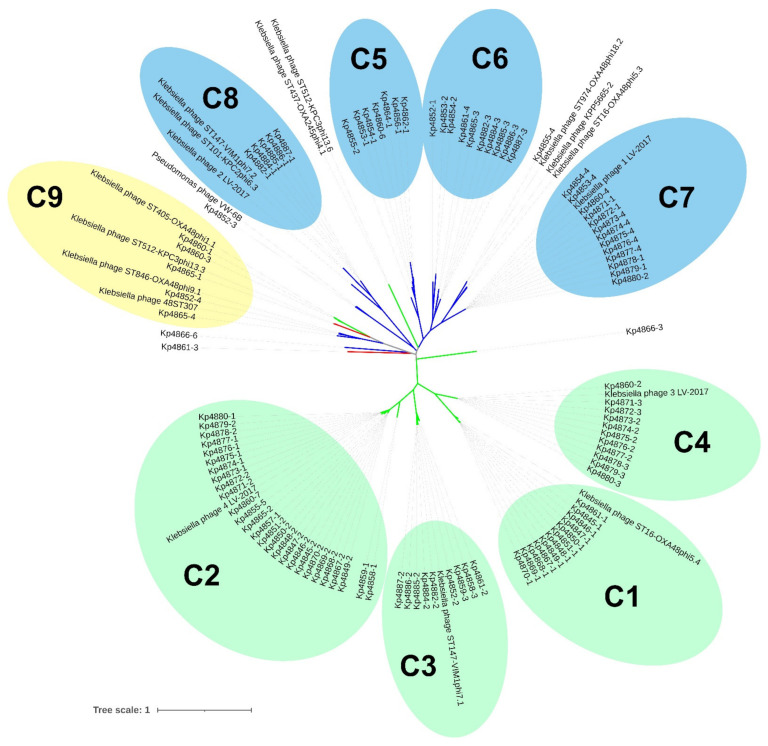
Phylogenetic tree of prophage genomic sequences. Tree was constructed using the Jukes–Cantor substitution model in PHYML 3.3.20180621 (Geneious Prime version 2021.1.1). Tree was analysed and annotated using Interactive Tree Of Life (iTOL) v6 [51]. Tree branches represent *Myoviridae* (green); *Siphoviridae* (blue); and *Podoviridae* (red). Shaded circles represent clusters with identities higher than 50%. *Myoviridae* (green); *Siphoviridae* (blue); and mixed cluster (yellow).

The similarity of prophage genomes was determined using an MAFFT alignment with default arguments and quantified as a heat-map matrix (Supplementary Figure S1). Whole-genome analysis revealed nine clusters of prophages with a genome identity above 50%, indicating strong evolutionary relationships. To understand the diversity of the prophage identified, a genomic phylogenetic tree was generated. Confirming our previous results, most of the prophages cluster by family group (Figure 4). Clusters C1-C4 and C5-C8 are comprised of sub-clusters containing highly related prophages (more than 70% identity), belonging to the *Myoviridae* and *Siphoviridae* families, respectively. Even for areas of lower identities, prophages tend to cluster according to family. Cluster C9 was revealed to be a mixed cluster with higher diversity, comprising prophages from *Myoviridae*, *Siphoviridae* and *Podoviridae* families, demonstrating that sub-clusters of the same family can be scattered in the phylogenetic tree and have an enormous genomic diversity.

### 3.5. Presence of Virulence Factors and Antibiotic Resistance Genes within K. pneumoniae Prophages

Prophages, even if defective, have implications on bacterial lifestyle, fitness, virulence, and the evolution of their bacterial host [21,25]. So, we searched for the presence of virulence factors and antimicrobial resistance genes encoded by the 150 prophages identified. Our analysis revealed the absence of any of the virulence or antimicrobial resistance-associated genes, using the available databases described in Materials and Methods. Since a rapid spreading of bacteria pathogenicity and an increase in host fitness is expected to be linked with prophages, we decided to analyse all prophage genomes and considered virulence genes to be those that might influence bacterial capacity to invade its host, evade or inhibit the host immune defense and survive and proliferate in different environmental conditions, in a similar way as described in Costa et al. (2018) [57]. By grouping the virulence and fitness genes in classes (Supplementary Table S4), we found that the TraR/DksA family transcription regulator was the most prevalent potential virulence factor. This family of transcriptional regulators is proposed to regulate a diverse set of genes, including those involved in virulence, the activation of stress response and providing indirect fitness advantages for the host [58,59]. Other potential virulence factors that may confer advantages to the bacteria-harboring the prophage were also found. These include the membrane-associated factor lipoprotein, which has been shown to play a direct role in virulence-associated functions, such as colonization, invasion, evasion of host defense, and immunomodulation [60]; the molecular chaperone DnaJ, that can have important functions in the assembly and replication of phage particles but may also be involved in bacterial motility and adhesion to the host, and has been described as essential for the virulence and colonization of *Streptococcus pneumoniae*, *P. aeruginosa* and *Salmonella* spp. [61,62,63]; UmuCD proteins, which are involved in persistence under stress conditions and already described in *K. pneumoniae* [64]; the serine/threonine phosphatase protein, an enzyme sensing and responding to environmental signals resulting from entering the host [65]; and the acetyltransferase family protein, which are enzymes indirectly involved in antibiotic, xenobiotic resistance and play a role in bacterial virulence [66].

Moreover, it was observed that the prophage Kp4852-1 had a closely adjacent T6SS-associated gene (ImpB protein) along the downstream regions of the prophage insertion site (Supplementary Figure S2, Supplementary Table S1). T6SS components belong to Type VI secretion system, which has been identified in several different pathogenic bacteria and appears to play different roles related to pathogenicity, host-range determination and/or niche adaptation [67,68].

### 3.6. Identification of Putative Endolysins within K. pneumoniae Prophages Genomes

Endolysins encoded by prophage genomes have attracted increased interest, particularly in the context of emerging antibiotic resistance. To study the nature of the endolysins encoded by prophages, our set of 150 *K. pneumoniae* prophages genomes, jointly with a set of 17 *Klebsiella* phagic genomes in GenBank, were analysed for putative endolysin identification (Supplementary Table S5). We were able to identify 132 endolysin sequences (115 endolysins from our dataset plus 17 endolysins from phages annotated), except for the *K. pneumoniae* intact prophage Kp4852-4 and 34 defective prophages, for which no endolysin was identified, pointing to the presence of cryptic prophages that are no longer able to pursue a lytic cycle. Endolysins were also present in some defective prophages, even if their sequences were still incomplete; such is the case of the prophages Kp4858-4, Kp4858-5, Kp4859-4 and Kp4859-5.

To determine the relationship and diversity of *K. pneumoniae* endolysins, we performed a genome and proteome heat map analysis of prophage sequences (Supplementary Figure S3). Genomic analysis revealed seven clusters of endolysins with genome identity above 60%, indicating strong evolutionary relationships. Interestingly, proteomic analysis revealed a diversity in the amino acid and nucleotide sequence, while still allowing the identification of the same seven clusters with an amino acid sequence identity above 40%, reinforcing precise function conservation [69].

**Figure 5:**
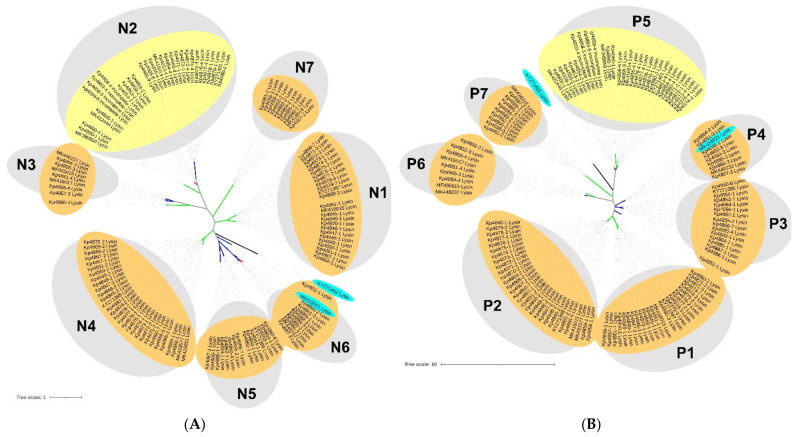
Phylogenetic trees of prophage endolysins based on (**A**) nucleotides and (**B**) amino acids sequences. Genomic tree was constructed using the Jukes–Cantor substitution model and the proteomic tree was constructed using the Le Gascuel substitution model in PHYML 3.3.20180621 (Geneious Prime version 2021.1.1). Trees were analysed and annotated using Interactive Tree Of Life (iTOL) v6 [51]. Tree branches represent *Myoviridae* (green); *Siphoviridae* (blue); *Podoviridae* (red); and *Ackermannviridae* (black) families (in silico determined). Grey-shaded circles represent clusters with identities higher than 50%. Small, shaded circles represent endolysins groups. Lysozymes/muramidases (orange); Chitinases (yellow); and Endopeptidases (light blue).

To understand if the endolysins clusters formed were related to a prophage family, we constructed genomic and proteomic phylogenetic trees and provided family information, as shown in Figure 5. Genomic clusters N1, N3-N5 and N7 (Figure 5a, also identified in Supplementary Figure S3a) have more than 60% nucleotides identity and are comprised of sub-clusters containing even highly related endolysins (>80% nucleotides identity). These sub-clusters are composed of endolysins that belong to prophages of the same family. Clusters N2 and N6 are composed of endolysins of different prophage families, although sharing a 65% and 45% nucleotide identity, respectively. Nevertheless, clusters of the same prophage family are scattered in the tree, demonstrating that endolysins of these prophage families can have significantly divergent genomes. A similar analysis was made when observing the proteomic tree (Figure 5b, also identified in Supplementary Figure S3b). Six clusters (clusters P1-P4 and P6-P7) of high endolysins proteome identity (>60% amino acids identity) are also clusters (clusters N1, N3-N7) with high genomic identity (>60% identity). Interestingly, cluster P5, which corresponds to genomic cluster N2, is a more diverse group, but still comprises related endolysins (>40% amino acids, > 45% nucleotides identity) and includes sub-clusters of highly related endolysins, with 95% amino acids and nucleotides identity belonging to different prophage families. Still, these results demonstrate a strong agreement between both analyses.

### 3.7. Classification of Endolysins within K. pneumoniae Prophages Genomes

On the basis of its structure homology, endolysins were assigned to a general classification (Supplementary Table S5), which were grouped according to sequence and structural homology in six groups: Group 1, endolysins related to P1 phage endolysin Lyz, which is an endolysin from *Escherichia coli* phage P1 (Bacteriophage P1) (EC:3.2.1.17); Group 2, endolysin R21 like-protein related to endolysin R21 from the *Enterobacteria* phage P21; Group 3, lysin β-1,4-N-acetylmuramidase related to lysozyme from bacteriophage lambda; Group 4, a group of chitinases (EC 3.2.1.14); Group 5, endopeptidases belonging to peptidase family C40; and Group 6, with other lysozymes which were not classified into the previous groups but which also function as lysozymes/muramidases.

The GO knowledgebase is the world’s largest source of information on the functions of genes. In this work, the gene ontology for phage endolysin was analysed using the QuickGo website (http://www.ebi.ac.uk/QuickGO/, last accessed July 2021), which is a web-based tool that allows easy browsing of the Gene Ontology (GO) provided by the GO Consortium annotation groups. Here, we searched three specific GO terms: lysozyme, chitinase and endopeptidase (Supplementary Figure S4). The GO term GO:0003796 corresponds to lysozyme activity, whose ontology by molecular function describes this protein as “Catalysis of the hydrolysis of the beta-(1->4) linkages between N-acetylmuramic acid and N-acetyl-D-glucosamine residues in a peptidoglycan”. The GO term GO:0004568 corresponds to chitinase activity, whose ontology by molecular function describes this protein as “Catalysis of the hydrolysis of (1->4)-beta linkages of N-acetyl-D-glucosamine (GlcNAc) polymers of chitin and chitodextrins”. For both, all the relationships in the ancestor chart are “Is a”, including the molecular function of hydrolase activity and peptidoglycan muralytic activity (Supplementary Figure S4a). The GO term GO:0004175 corresponds to endopeptidase activity, whose ontology by molecular function describes this protein as “Catalysis of the hydrolysis of internal, alpha-peptide bonds in a polypeptide chain”. For both lysozyme activity (GO:0003796) and chitinase activity (GO:0004568), all the relationships in the Ancestor Chart are “Is a”, including the molecular function of hydrolase activity and peptidoglycan muralytic activity (Supplementary Figure S4b). For endopeptidase activity (GO:0004175), most of the relationships are “Is a”, including the molecular function of peptidase activity and catalytic activity, acting on a protein and hydrolase activity; but it is also possible to see one “Part of”, showing the biological process of proteolysis (Supplementary Figure S4c).

Joining previous analysis, structural homology and GO terms searched, endolysins were attributed to three general classes: lysozyme/muramidases (*n* = 99), glycosidases/chitinases (*n* = 31) and endopeptidases (*n* = 2). The lysozyme and chitinase classes were identified in all *K. pneumoniae* prophages described here, whereas the two endopeptidases were identified in two related phages (*Klebsiella* phages ST846-OXA48phi9.1 and 48ST307; GenBank: MK416021.1 and KY271402.1, respectively).

Although endolysins included into Group 1–5 have different nucleotides and amino acid sequences, they fall into the same class. All of them are lysozymes/muramidases and can hydrolyse β-1,4 linkages between N-acetylmuramic acid and N-acetyl-D-glucosamine residues in peptidoglycan as well as inside N-acetyl-D-glucosamine residues. In the case of the chitinases (Group 4), the enzyme binds to chitin and randomly cleaves glycosidic linkages in chitin and chitodextrins in a non-processive mode, generating chitooligosaccharides and free ends on which exo-chitinases and exo-chitodextrinases can act [70]. Remarkably, Group 6 includes one endopeptidase identified from *Klebsiella* phage ST846-OXA48phi9.1 (GenBank: MK416021), which share a higher identity (higher than 80%) with endolysin R21 like-proteins despite their different function. The endolysins identified here had a predicted molecular weight between 16.06 and 24.31 kDa, consistent with the molecular weight described in the literature for phages of gram-negative bacteria [71].

## 4. Discussion

The spread of highly virulent and antibiotic-resistant *K. pneumoniae* strains, both in hospitals and natural environments, requires more knowledge about *Klebsiella* prophages as mediators of gene transfer often offering advantageous features to the host, as well as their antibacterial potential (including gene-encoded products involved in host lysis called endolysins). Particular attention has been given to phages and endolysins which demonstrate activity on highly virulent and multidrug-resistant pathogens including *K. pneumoniae* [32].

In this study, we analysed 40 recently sequenced *K. pneumoniae* clinical strains and found prophages in all genomes. Almost all genomes harbored more than one prophage, consistent with the fact that *K. pneumoniae* is one of the species with more prophages among widely sequenced bacteria [72,73], suggesting that prophages are important for its biology. Since prophages are involved in the transduction of genetic material horizontally, the presence of the same prophages in different isolates indicates their horizontal movement and importance in genomic plasticity or evolution [74]. Most prophages were intact (70.9%), which may indicate a recent integration, and 29.1% were defective (incomplete or questionable). Incomplete and questionable prophages often lack essential phage functions [73] and therefore our further analysis was focused on intact prophages. Only a few studies have characterized the prevalence of prophages in *K. pneumoniae* species, although they are genetic elements that significantly contribute to genome variability, evolution, and virulence of their bacterial hosts [32,55,56,72,73,75].

In our analysis, the size of *K. pneumoniae* prophage genomes varied from 8.9 to 60.8 kbp, with an average of 37.4 kbp, which agrees with the literature for what has already been described for *K. pneumoniae* and enterobacteria [73,75,76]. Using an in silico approach, among the 104 intact prophages, we found *Myoviridae* to be the most represented family (59.6%), followed by *Siphoviridae* (38.5%) and *Podoviridae* (1.9%). The same distribution was observed in other studies [55,56]. *Myoviridae*, which are typically the largest phage family, had size genomes below average, while *Siphoviridae* had sizes slightly above the average. PHASTER analysis of draft genomes (especially if these were distributed through different contigs) could erroneously delimit prophages, for which all prophage sequences analysed were manually curated. Although we have manually curated the prophage insertion sites and scaffolded prophages that were split in several contigs, this may result in unexpected or variable genome sizes. These differences may also result from the acquisition of bacterial genes adjacent to the prophage during repeated excision and integration cycles or loss and genetic degradation that caused the reduction of its genome size [76].

Prophages of *K. pneumoniae* were found to be greatly similar. Our comparison of 104 intact prophages revealed that some prophages shared more than 50% genome identity, indicating strong evolutionary relationships. Comparing our prophage genomes against public databases, we found 17 *Klebsiella* phages which share similarity with the 104 intact prophages in terms of query coverage and identity (in some cases higher than 60%) and this helped us to identify nine clusters composed by identical prophages. Moreover, related clusters tend to group prophages of the same family. However, at same time, the prophages were also greatly diversified. A comparison of these nine clusters revealed less than 30% of genome identity and we found a *Pseudomonas* phage VW-6B that shares an identity higher than 31% with one of the prophages identified here. This may indicate that the common ancestor of these two species was infected by a phage that co-evolved with the host bacteria during speciation, or by phage transfer between species. 

*K. pneumoniae* prophages have an open pan-genome, meaning that for each new prophage genome added, new genes contribute for the pan-genome. Thus, the inclusion of more prophages is expected to raise the number of the pan-genome size of 892 genes so far determined, which is also corroborated by the high percentage of singleton genes (43.6%, 389/892). On the other hand, the reduced number of core genes points to high sequence diversity, only preserving essential structural genes. 

Some prophages carry genes that can alter the features of the host, ranging from increased host fitness to increased virulence, and many studies have reported the connection of the pathogen virulence to the acquisition of prophages [21,77]. In fact, even defective prophages are considered as potential mobile elements carrying virulence factors [25]. Thus, although open-access research tools did not find virulence factors, a detailed analysis showed otherwise, revealing several potential virulence factors that can be related to bacteria fitness and influence the ability of the bacterium to colonize its host and survive in adverse environments. 

Prophages and their bacterial hosts have common evolutionary interests since the proliferation of the host also results in increased prophage population. Thus, some prophages provide the bacterium beneficial traits, such as increased fitness, and confer new virulence factors and/or antibiotic resistance genes exploited for bacterial pathogenesis [25,76]. Accordingly, we identified several putative virulence factors, such as TraR/DksA family transcriptional regulator, membrane-associated lipoprotein, molecular chaperone DnaJ and other proteins with functions in persistence under stress conditions, interaction with host cells and regulation of virulence gene expression. TraR family regulators may also play a role in prophage propagation by interfering with the host mechanisms of regulation, increasing the bacterial conjugation, and improving the transmission of the prophage by lateral gene transfer between bacteria [59]. Taken together, *K. pneumoniae* may benefit from carrying a prophage due to the putative beneficial genes carried by them. Thus, *K. pneumoniae* prophages may confer an evolutionary fitness benefit to the host due to the presence of virulence factors, which should be further study. On the other hand, our study confirmed the previous description by Perdigão et al. (2020), where patterns of resistance-conferring genes related to antimicrobial resistance were associated with chromosomal mutations and plasmid mobilization [35]. The prophages sequences described here did not contain the genes responsible by antibiotic resistance in these *K. pneumoniae* isolates. 

Prophages have coevolved with bacteria for more than a billion years and have developed efficient strategies to lyse and thus kill their bacterial host at the end of the lytic cycle for progeny release [16]. Endolysins are proteins used in this lytic process and have been used in many scientific works for the development of antibacterial therapeutics [71,78,79,80]. Depending on cleavage sites, they can be categorized into four different groups: (a) N-acetylmuramidases (lysozymes), (b) N-acetyl-β-D-glucosaminidases (glycosidases) (c) N-acetylmuramoyl-L-alanine amidases and (d) endopeptidases [71,81]. In this study, we exploited the *K. pneumoniae* sequenced genomes to identify endolysins in their prophages and characterized them. We identified 132 endolysins (115 endolysins from our prophage genomes and 17 lysins harbored by the most related phages and the endolysins were assigned to three groups: lysozymes/muramidases, glycosidases/chitinases and endopeptidases. The group of lysozymes included endolysins related to endolysin LyzP1 and R21, which are two lysozymes of phages P1 and 21, respectively, and were the first endolysins showing a signal-anchor-release (SAR) domain [82]. In contrast to phage lysozymes like T4, which accumulate a fully folded and enzymatically active endolysin in the cytoplasm, SAR endolysins are the first endolysins secreted as enzymatically inactive form anchored to the membrane by the N-terminal SAR domain to avoid premature lysis of the infected host [83]. The group of chitinases are members of glycoside hydrolase (GH) family 18 and 19 and are also a broad, lysozyme-like superfamily cleaving chitin, which is the second most abundant biopolymer on the planet and is a linear, insoluble homopolymer composed of β-1,4 linked subunits of N-acetyl glucosamine polymers, a structure uncommon in bacterial cell walls. Most of the bacterial chitinases isolated and sequenced so far are included in GH family 18, have a molecular weight range of 20–60 kDa and are smaller than plant chitinases (40–85 kDa) [70], which agrees with the molecular weight predicted here. The third group included endopeptidases, but this group was only identified in the related *Klebsiella* phages, 48ST307 and ST846-OXA48phi9.1.

Our study shows that the diversity of *K. pneumoniae* prophages is vast and continues to expand. In addition, this genome analysis serves as a basis for the characterization and evolutionary relationship of prophages harbored by *K. pneumoniae* and identification of relevant proteins, such as endolysins, which may have biomedical applications for new and protein-based antimicrobials. Future work will focus on cloning and characterizing *K. pneumoniae* endolysins and studying their lysis activity and potential as bactericidal products.

## Figures and Tables

**Table 1:** Intact prophage genomes characterization. Features such as patient, GC%, length, CDS, cluster, family (in silico-determined) and most related-phage identified are shown for each prophage. GC, guanine–cytosine.

			Prophages									
Patients	Strains	Prophages	GC%	Length (kbp)	CDS	Cluster	Family	Related Phages	Accession Number	Query Cover	E Value	Per. Ident
Patient 1	Kp4845	PKp4845-1	50,4	33,4	44	C1	*Myoviridae*	*Klebsiella* phage ST16-OXA48phi5.4	MK416015.1	75%	0.0	96.19%
		PKp4845-2	50,4	33,4	44	C2		*Klebsiella* phage 4 LV-2017	KY271398.1	70%	0.0	97.67%
	Kp4846	PKp4846-1	50,4	33,4	44	C1		*Klebsiella* phage ST16-OXA48phi5.4	MK416015.1	75%	0.0	96.19%
		PKp4846-2	50,4	33,4	44	C2		*Klebsiella* phage 4 LV-2017	KY271398.1	70%	0.0	97.67%
	Kp4847	PKp4847-1	50,4	33,4	44	C1		*Klebsiella* phage ST16-OXA48phi5.4	MK416015.1	75%	0.0	96.19%
		PKp4847-2	50,4	33,4	44	C2		*Klebsiella* phage 4 LV-2017	KY271398.1	70%	0.0	97.67%
	Kp4848	PKp4848-1	50,4	33,4	44	C1		*Klebsiella* phage ST16-OXA48phi5.4	MK416015.1	75%	0.0	96.19%
		PKp4848-2	50,4	33,4	44	C2		*Klebsiella* phage 4 LV-2017	KY271398.1	70%	0.0	97.67%
	Kp4850	PKp4850-1	50,4	33,4	44	C1		*Klebsiella* phage ST16-OXA48phi5.4	MK416015.1	75%	0.0	96.19%
		PKp4850-2	51,2	32,3	43	C2		*Klebsiella* phage 4 LV-2017	KY271398.1	70%	0.0	97.67%
	Kp4851	PKp4851-1	48,3	46,7	55	C1		*Klebsiella* phage ST16-OXA48phi5.4	MK416015.1	75%	0.0	96.19%
		PKp4851-2	50,1	35,6	47	C2		*Klebsiella* phage 4 LV-2017	KY271398.1	70%	0.0	97.67%
Patient 2	Kp4852	PKp4852-1	50,1	35,6	47	C6	*Myoviridae*	*Klebsiella* phage ST101-KPC2phi6.3	MK416017.1	35%	0.0	95.09%
		PKp4852-2	50,1	35,6	47	C3		*Klebsiella* phage ST147-VIM1phi7.1	MK416018.1	56%	0.0	96.97%
		PKp4852-3	50,1	35,6	47	N/D		*Pseudomonas* phage VW-6B	MF975721.1	31%	0.0	77.91%
		PKp4852-4	50,1	35,6	47	C9		*Klebsiella* phage ST846-OXA48phi9.1	MK416021.1	77%	0.0	98.39%
Patient 3	Kp4853	PKp4853-1	50,1	35,6	47	C5	*Myoviridae*	*Klebsiella* phage 2 LV-2017	KY271396.1	54%	0.0	94.42%
		PKp4853-2	50,1	35,6	47	C6						
		PKp4853-4	50,1	35,6	47	C7		*Klebsiella* phage 1 LV-2017	KY271401.1	74%	0.0	99.95%
	Kp4854	PKp4854-1	50,1	35,6	47	C5		*Klebsiella* phage 2 LV-2017	KY271396.1	54%	0.0	94.42%
		PKp4854-2	50,1	35,6	47	C6						
		PKp4854-4	50,1	35,6	47	C7		*Klebsiella* phage 1 LV-2017	KY271401.1	74%	0.0	99.95%
Patient 4	Kp4855	PKp4855-2	50,1	35,6	47	C5	*Myoviridae*	*Klebsiella* phage ST16-OXA48phi5.3	MK416014.1	28%	0.0	96.13%
		PKp4855-4	51,1	31,9	43	N/D		*Klebsiella* phage ST974-OXA48phi18.2	MK448237.1	68%	0.0	96.79%
		PKp4855-5	51,1	31,9	43	C2		*Klebsiella* phage 4 LV-2017	KY271398.1	82%	0.0	96.43%
Patient 5	Kp4856	PKp4856-1	51,1	35,1	44	C5	*Myoviridae*	*Klebsiella* phage 2 LV-2017	KY271396.1	46%	0.0	97.25%
Patient 6	Kp4857	PKp4857-1	51,1	35,1	44	C2	*Myoviridae*	*Klebsiella* phage 4 LV-2017	KY271398.1	70%	0.0	97.67%
Patient 7	Kp4858	PKp4858-1	51,1	35,1	44	C2	*Myoviridae*	*Klebsiella* phage ST512-KPC3phi13.6/*Klebsiella* phage ST437-OXA245phi4.1	MK433577.1/MK416011.1	86%	0.0	97.25%
		PKp4858-3	51,1	35,1	44	C3		*Klebsiella* phage ST147-VIM1phi7.1	MK416018.1	64%	0.0	95.28%
Patient 8	Kp4859	PKp4859-1	51,1	35,1	44	C2	*Myoviridae*	*Klebsiella* phage ST512-KPC3phi13.6/*Klebsiella* phage ST437-OXA245phi4.1	MK433577.1/MK416011.1	86%	0.0	97.25%
		PKp4859-3	51	34,1	40	C3		*Klebsiella* phage ST147-VIM1phi7.1	MK416018.1	64%	0.0	95.28%
Patient 9	Kp4860	PKp4860-1	52,2	29,7	41	C9	*Myoviridae*	*Klebsiella* phage ST405-OXA48phi1.1	MK388859.1	88%	0.0	85.05%
		PKp4860-2	52,2	29,7	41	C4		*Klebsiella* phage 3 LV-2017	KY271397.1	100%	0.0	100%
		PKp4860-3	51,3	33,5	43	C9		*Klebsiella* phage ST405-OXA48phi1.1	MK388859.1	80%	0.0	86.57%
		PKp4860-4	50,2	36,7	43	C7		*Klebsiella* phage 1 LV-2017	KY271401.1	74%	0.0	99.95%
		PKp4860-6	50,2	36,7	43	C5		*Klebsiella* phage 2 LV-2017	KY271396.1	36%	0.0	93.97%
		PKp4860-7	50,2	36,7	43	C2		*Klebsiella* phage 4 LV-2017	KY271398.1	100%	0.0	100%
Patient 10	Kp4861	PKp4861-1	50,2	36,7	43	C1	*Myoviridae*	*Klebsiella* phage ST16-OXA48phi5.4	MK416015.1	74%	0.0	96.13%
		PKp4861-2	50,2	36,7	43	C3		*Klebsiella* phage ST147-VIM1phi7.1	MK416018.1	55%	0.0	97.6%
		PKp4861-3	50,2	36,7	43	N/D						
		PKp4861-4	50,2	36,7	43	C6		*Klebsiella* phage 1 LV-2017	KY271401.1	31%	0.0	96.90%
Patient 11	Kp4862	PKp4862-1	50,2	36,7	43	C5	*Myoviridae*	*Klebsiella* phage 2 LV-2017	KY271396.1	46%	0.0	97.25%
Patient 13	Kp4864	PKp4864-1	50,2	36,7	43	C5	*Myoviridae*	*Klebsiella* phage 2 LV-2017	KY271396.1	35%	0.0	85.93%
Patient 14	Kp4865	PKp4865-1	50,2	36,7	43	C9	*Myoviridae*	*Klebsiella* phage ST512-KPC3phi13.3	MK422448.1	73%	0.0	97.23%
		PKp4865-2	50,2	36,7	43	C2		*Klebsiella* phage 4 LV-2017	KY271398.1	53%	0.0	98.48%
		PKp4865-3	49,9	37	41	C6		*Klebsiella* phage KPP5665-2	MF695815.1	25%	0.0	93.71%
		PKp4865-4	54,4	35	48	C9		*Klebsiella* phage 48ST307	KY271402.1	28%	0.0	94.55%
Patient 15	Kp4866	PKp4866-3	54,4	35	48	N/D	*Myoviridae*					
		PKp4866-6	54,4	35	48	N/D						
	Kp4867	PKp4867-1	54,4	35	48	C1		*Klebsiella* phage ST16-OXA48phi5.4	MK416015.1	75%	0.0	96.19%
		PKp4867-2	54,4	35	48	C2		*Klebsiella* phage 4 LV-2017	KY271398.1	70%	0.0	97.67%
	Kp4868	PKp4868-1	54,4	35	48	C1		*Klebsiella* phage ST16-OXA48phi5.4	MK416015.1	75%	0.0	96.19%
		PKp4868-2	54,4	35	48	C2		*Klebsiella* phage 4 LV-2017	KY271398.1	70%	0.0	97.67%
	Kp4870	PKp4870-1	54,4	35	48	C1		*Klebsiella* phage ST16-OXA48phi5.4	MK416015.1	75%	0.0	96.19%
		PKp4870-2	54,4	35	48	C2		*Klebsiella* phage 4 LV-2017	KY271398.1	70%	0.0	97.67%
Patient 16	Kp4871	PKp4871-1	50,6	39,1	63	C7	*Siphoviridae*	*Klebsiella* phage 1 LV-2017	KY271401.1	74%	0.0	99.95%
		PKp4871-2	50,6	39,1	63	C2		*Klebsiella* phage 4 LV-2017	KY271398.1	100%	0.0	100%
		PKp4871-3	50,6	39,1	63	C4		*Klebsiella* phage 3 LV-2017	KY271397.1	100%	0.0	100%
Patient 17	Kp4872	PKp4872-1	50,6	39,1	63	C7	*Siphoviridae*	*Klebsiella* phage 1 LV-2017	KY271401.1	74%	0.0	99.95%
		PKp4872-2	50,6	39,1	63	C2		*Klebsiella* phage 4 LV-2017	KY271398.1	100%	0.0	100%
		PKp4872-3	52	40,9	57	C4	*Podoviridae*	*Klebsiella* phage 3 LV-2017	KY271397.1	100%	0.0	100%
	Kp4873	PKp4873-1	50,2	45,9	51	C2	*Siphoviridae*	*Klebsiella* phage 4 LV-2017	KY271398.1	100%	0.0	100%
		PKp4873-2	53,2	34,9	32	C4		*Klebsiella* phage 3 LV-2017	KY271397.1	100%	0.0	100%
		PKp4873-4	55	33,1	45	C7	*Myoviridae*	*Klebsiella* phage 1 LV-2017	KY271401.1	74%	0.0	99.95%
	Kp4874	PKp4874-1	50,4	39,4	49	C2	*Siphoviridae*	*Klebsiella* phage 4 LV-2017	KY271398.1	100%	0.0	100%
		PKp4874-2	50,5	39,5	49	C4		*Klebsiella* phage 3 LV-2017	KY271397.1	100%	0.0	100%
		PKp4874-4	50,5	39,5	49	C7		*Klebsiella* phage 1 LV-2017	KY271401.1	74%	0.0	99.95%
	Kp4875	PKp4875-1	50,5	39,5	49	C2		*Klebsiella* phage 4 LV-2017	KY271398.1	100%	0.0	100%
		PKp4875-2	50,5	39,5	49	C4		*Klebsiella* phage 3 LV-2017	KY271397.1	100%	0.0	100%
		PKp4875-4	50,5	39,5	49	C7		*Klebsiella* phage 1 LV-2017	KY271401.1	74%	0.0	99.95%
	Kp4876	PKp4876-1	50,5	39,5	49	C2		*Klebsiella* phage 4 LV-2017	KY271398.1	100%	0.0	100%
		PKp4876-2	50,5	39,5	49	C4		*Klebsiella* phage 3 LV-2017	KY271397.1	100%	0.0	100%
		PKp4876-4	50,5	39,5	49	C7		*Klebsiella* phage 1 LV-2017	KY271401.1	74%	0.0	99.95%
Patient 18	Kp4877	PKp4877-1	50,5	39,5	49	C2	*Siphoviridae*	*Klebsiella* phage 4 LV-2017	KY271398.1	100%	0.0	100%
		PKp4877-2	50,5	39,5	49	C4		*Klebsiella* phage 3 LV-2017	KY271397.1	100%	0.0	100%
		PKp4877-4	50,5	39,5	49	C7		*Klebsiella* phage 1 LV-2017	KY271401.1	74%	0.0	99.95%
Patient 19	Kp4878	PKp4878-1	50,4	39,7	49	C7	*Siphoviridae*	*Klebsiella* phage 1 LV-2017	KY271401.1	74%	0.0	99.95%
		PKp4878-2	50,4	40,1	50	C2		*Klebsiella* phage 4 LV-2017	KY271398.1	100%	0.0	100%
		PKp4878-3	50,4	40,1	50	C4		*Klebsiella* phage 3 LV-2017	KY271397.1	100%	0.0	100%
	Kp4879	PKp4879-1	50,4	40,1	50	C7		*Klebsiella* phage 1 LV-2017	KY271401.1	74%	0.0	99.95%
		PKp4879-2	50,4	40,1	50	C2		*Klebsiella* phage 4 LV-2017	KY271398.1	100%	0.0	100%
		PKp4879-3	50,4	40,1	50	C4		*Klebsiella* phage 3 LV-2017	KY271397.1	100%	0.0	100%
	Kp4880	PKp4880-1	49,7	39,3	55	C2		*Klebsiella* phage 4 LV-2017	KY271398.1	100%	0.0	100%
		PKp4880-2	50,4	41,5	61	C7		*Klebsiella* phage 1 LV-2017	KY271401.1	74%	0.0	99.95%
		PKp4880-3	50,6	38,6	56	C4		*Klebsiella* phage 3 LV-2017	KY271397.1	100%	0.0	100%
Patient 21	Kp4882	PKp4882-1	51,4	39,8	67	C8	*Siphoviridae*	*Klebsiella* phage ST101-KPC2phi6.3	MK416017.1	23%	0.0	97.40%
		PKp4882-2	50,9	42,2	58	C3		*Klebsiella* phage ST147-VIM1phi7.1	MK416018.1	70%	0.0	100.00%
		PKp4882-3	50,9	42,2	58	C6		*Klebsiella* phage ST147-VIM1phi7.2	MK448232.1	85%	0.0	99.99%
Patient 23	Kp4884	PKp4884-1	49,8	45,5	60	C8	*Siphoviridae*	*Klebsiella* phage ST101-KPC2phi6.3	MK416017.1	23%	0.0	97.40%
		PKp4884-2	51,2	50,6	77	C3		*Klebsiella* phage ST147-VIM1phi7.1	MK416018.1	70%	0.0	100.00%
		PKp4884-3	52,1	48,6	75	C6		*Klebsiella* phage ST147-VIM1phi7.2	MK448232.1	85%	0.0	99.99%
	Kp4885	PKp4885-1	52,1	48,6	75	C8		*Klebsiella* phage ST101-KPC2phi6.3	MK416017.1	23%	0.0	97.40%
		PKp4885-2	51,5	46,8	78	C3		*Klebsiella* phage ST147-VIM1phi7.1	MK416018.1	70%	0.0	100.00%
		PKp4885-3	53	46,9	68	C6		*Klebsiella* phage ST147-VIM1phi7.2	MK448232.1	85%	0.0	99.99%
Patient 24	Kp4886	PKp4886-1	53	47,1	68	C8	*Siphoviridae*	*Klebsiella* phage ST101-KPC2phi6.3	MK416017.1	23%	0.0	97.40%
		PKp4886-2	52,7	48,4	73	C3		*Klebsiella* phage ST147-VIM1phi7.1	MK416018.1	70%	0.0	100.00%
		PKp4886-3	54,9	30,5	45	C6	*Myoviridae*	*Klebsiella* phage ST147-VIM1phi7.2	MK448232.1	85%	0.0	99.99%
Patient 25	Kp4887	PKp4887-1	54,9	30,2	45	C8	*Myoviridae*	*Klebsiella* phage ST101-KPC2phi6.3	MK416017.1	23%	0.0	97.40%
		PKp4887-2	54	35,2	43	C3	*Siphoviridae*	*Klebsiella* phage ST147-VIM1phi7.1	MK416018.1	70%	0.0	100.00%
		PKp4887-3	50,9	40,2	53	C6	*Podoviridae*	*Klebsiella* phage ST147-VIM1phi7.2	MK448232.1	85%	0.0	99.99%
Patient 26	Kp4849	PKp4849-1	50,4	33,4	44	C1	*Myoviridae*	*Klebsiella* phage ST16-OXA48phi5.4	MK416015.1	75%	0.0	96.19%
		PKp4849-2	50,4	33,4	44	C2		*Klebsiella* phage 4 LV-2017	KY271398.1	70%	0.0	97.67%
	Kp4869	PKp4869-1	54,4	35	48	C1		*Klebsiella* phage ST16-OXA48phi5.4	MK416015.1	75%	0.0	96.19%
		PKp4869-2	54,4	35	48	C2		*Klebsiella* phage 4 LV-2017	KY271398.1	70%	0.0	97.67%

## Data Availability

Data are contained within the article or supplementary data. Intact prophage sequences were deposit in GenBank under the accession OK490394-OK490474.

## Supplementary Materials

The following are available online at https://www.mdpi.com/article/10.3390/microorganisms9112252/s1. Figure S1: MAFFT alignment of whole 104 genomic prophage sequences from *K. pneumoniae*. Annotated prophages which share homology with our sequences were included. Darker zones indicate higher identity. Clusters of prophages with identities higher than 50% are indicated and numbered. *Myoviridae*, green; *Siphoviridae*, blue; and mixed-cluster, yellow; Figure S2: Genomic feature of the intact prophage Kp4852-1 in relation to its Type VI Secretion System component (blue) along the downstream regions of the prophage insertion site; Figure S3: MAFFT alignment of 115 prophage endolysins (A) nucleotides and (B) amino acids sequences extracted from *K. pneumoniae* prophage sequences and 17 phage endolysins sequences extracted from *Klebsiella* which share homology with our sequences. Darker zones indicate higher identity: black, 90%; dark-grey, 70%, grey, 50%, and light-grey, 30%; Figure S4: Ancestry chart for (A) lysozyme activity (GO:0003796), (B) chitinase activity (GO:0004568) and (C) endopeptidase activity (GO:0004175). Table S1. Genomic details of the 150 identified prophages; Table S2: Prophages classification (intact, incomplete, questionable). GC, guanine–cytosine. Defective, incomplete + questionable; Table S3: *K. pneumoniae* prophages pan-genome determined with Roary for a protein identity of 40%. Prophage genes present in more than half of the genomes are in bold; Table S4: Virulence and fitness factors identified for 150 prophages of *K. pneumoniae* strains; Table S5: Endolysins characterization.
