# Supplementary material for: Genomic Analysis of Prophages from Klebsiella pneumoniae Clinical Isolates
Source: Microorganisms. 2021 Oct 28;9(11):2252. doi: 10.3390/microorganisms9112252 (PMC8617712; doi:10.3390/microorganisms9112252)

Supplementary Figure S1. MAFFT alignment of whole 104 genomic prophage sequences from *K. pneumoniae*. Annotated prophages which share homology with our sequences were included. Darker zones indicate higher identity. Clusters of prophages with identities higher than 50% are indicated and numbered. *Myoviridae*, green; *Siphoviridae*, blue; mixed-cluster, yellow.

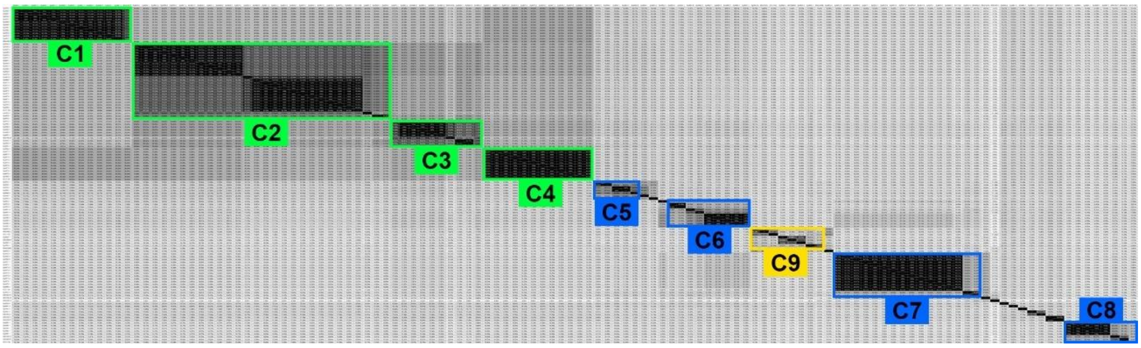

Supplementary Figure S2. Genomic feature of the intact prophage Kp4852-1 in relation to its Type VI Secretion System component (blue) along the downstream regions of the prophage insertion site.

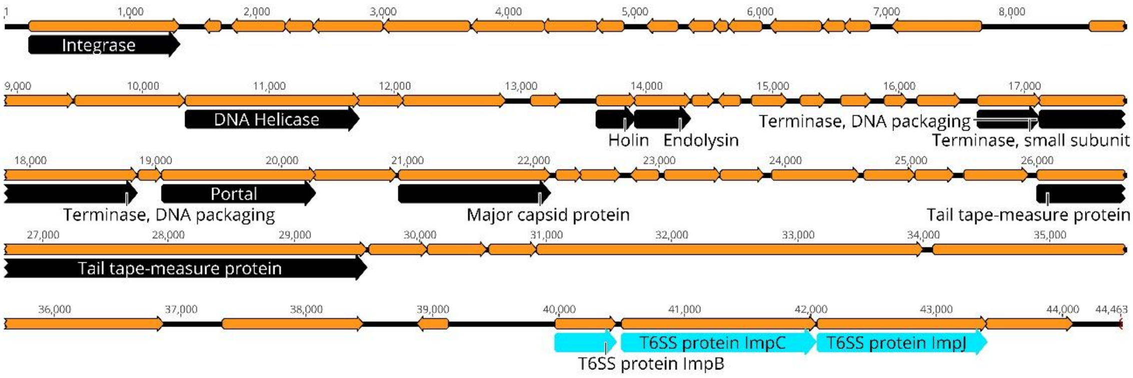

Supplementary Figure S3. MAFFT alignment of 115 prophage endolysins (A) nucleotides and (B) amino acids sequences extracted from *K. pneumoniae* prophage sequences and 17 phage endolysins sequences extract from *Klebsiella* which share homology with our sequences. Darker zones indicate higher identity: black, 90%; dark-grey, 70%, grey, 50%, light-grey, 30%

A.

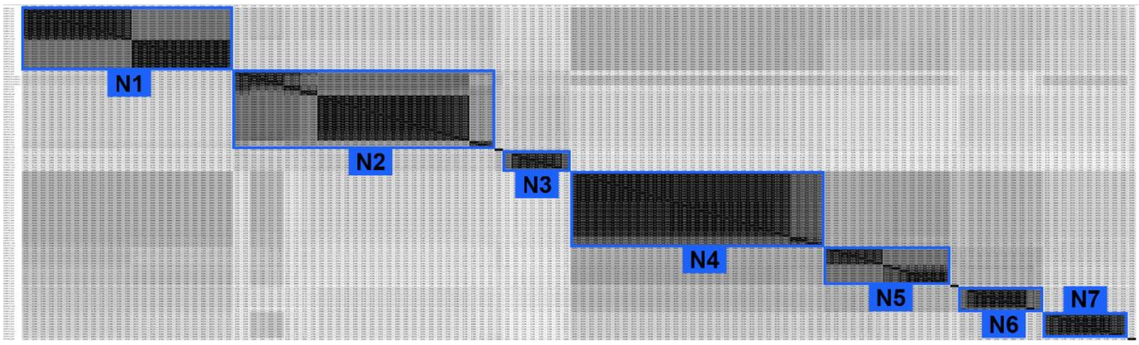

B.

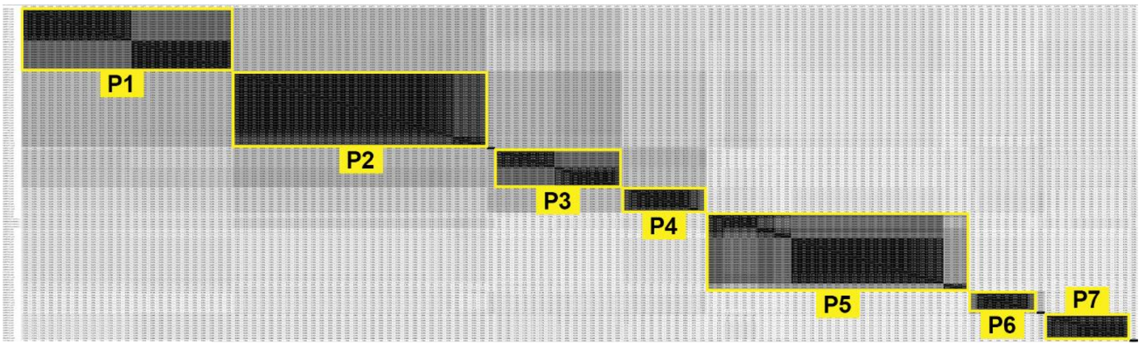

Supplementary Figure S4. Ancestry chart for (A) lysozyme activity (GO:0003796), (B) chitinase activity (GO:0004568) and (C) endopeptidase activity (GO:0004175).

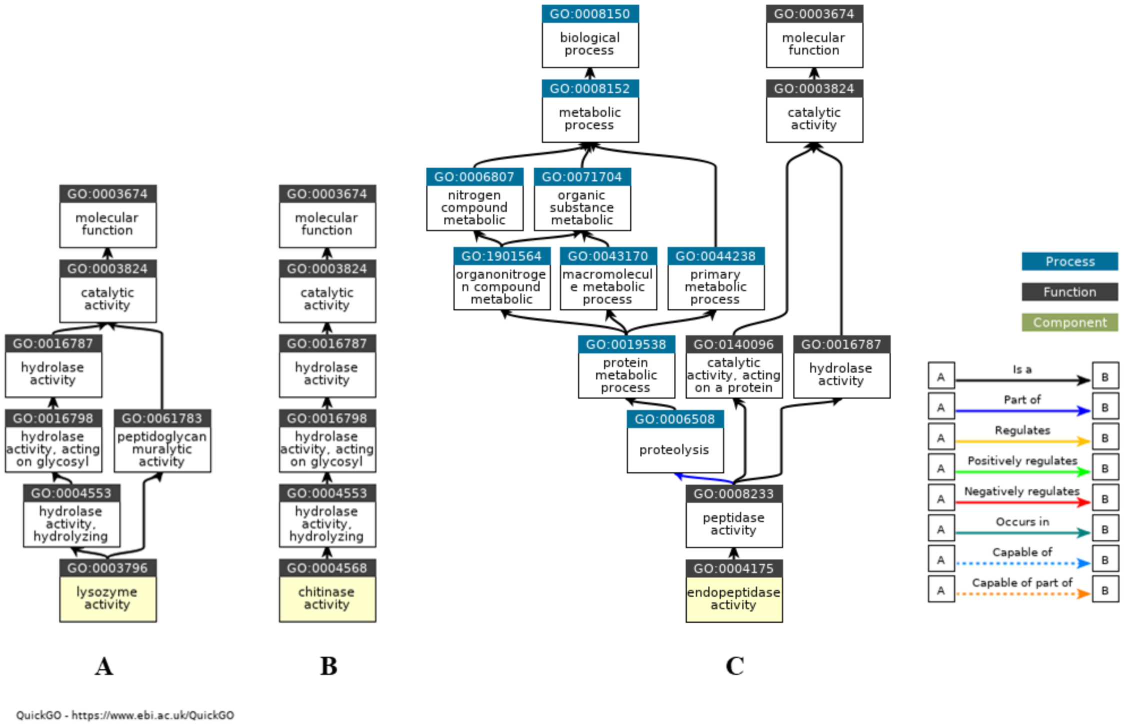

Supplement: Supplementary file 1 [file microorganisms-09-02252-s001.zip › Manuscript_Supplementary Figures.pdf]
